# Supplementary material for: From histology to macroscale function in the human amygdala
Source: eLife. 2025 Feb 13;13:RP101950. doi: 10.7554/eLife.101950 (PMC11825128; doi:10.7554/eLife.101950)
Supplement: Supplementary file 1. [file elife-101950-supp1.docx]

**Supplementary file 1a.** Correlation values between UMAP parameter U1 and features.

| Kernel size: | 2 | 4 | 6 | 8 | 10 |
| --- | --- | --- | --- | --- | --- |
| Mean | 0.850 | 0.909 | 0.938 | 0.954 | 0.962 |
| Variance | -0.010 | 0.045 | 0.081 | 0.104 | 0.118 |
| Skewness | -0.238 | -0.223 | -0.205 | -0.195 | -0.196 |
| Kurtosis | 0.311 | 0.369 | 0.389 | 0.401 | 0.408 |

**Supplementary file 1b.** Correlation values between UMAP parameter U2 and features.

| Kernel size: | 2 | 4 | 6 | 8 | 10 |
| --- | --- | --- | --- | --- | --- |
| Mean | -0.011 | -0.021 | -0.029 | -0.035 | -0.038 |
| Variance | 0.399 | 0.493 | 0.537 | 0.555 | 0.555 |
| Skewness | 0.396 | 0.586 | 0.664 | 0.693 | 0.684 |
| Kurtosis | 0.260 | 0.441 | 0.503 | 0.509 | 0.481 |

**Supplementary file 1c.** U1 correlations (r) with all 3 axes across all subjects and BigBrain **(*p_null_* < 0.05).**

|  | S1 | S2 | S3 | S4 | S5 | S6 | S7 | S8 | S9 | S10 | BB |
| --- | --- | --- | --- | --- | --- | --- | --- | --- | --- | --- | --- |
| I-F | **0.71** | **0.77** | **0.65** | **0.54** | **0.69** | **0.71** | **0.70** | **0.57** | **0.86** | **0.68** | **0.82** |
| P-A | 0.11 | **0.31** | 0.01 | 0.06 | 0.02 | 0.02 | 0.18 | 0.15 | 0.10 | 0.10 | **0.51** |
| M-L | **0.77** | **0.47** | **0.59** | **0.66** | **0.61** | **0.80** | **0.77** | **0.67** | **0.68** | **0.85** | 0.15 |

**Supplementary file 1d.** U2 correlations (r) with all 3 axes across all subjects and BigBrain **(*p_null_* < 0.05).**

|  | S1 | S2 | S3 | S4 | S5 | S6 | S7 | S8 | S9 | S10 | BB |
| --- | --- | --- | --- | --- | --- | --- | --- | --- | --- | --- | --- |
| I-F | **0.56** | 0.11 | 0.29 | **0.52** | **0.35** | **0.58** | 0.15 | 0.13 | 0.13 | 0.14 | 0.02 |
| P-A | 0.14 | 0.10 | **0.34** | **0.26** | 0.16 | **0.47** | **0.50** | **0.43** | **0.29** | **0.20** | **0.25** |
| M-L | 0.24 | 0.04 | 0.08 | 0.21 | **0.27** | 0.25 | **0.58** | **0.55** | 0.19 | **0.70** | 0.22 |
